# Plasma-free hemoglobin and microvascular response to fresh or old blood transfusion in septic patients

**DOI:** 10.1186/cc13291

**Published:** 2014-03-17

**Authors:** A Donati, E Damiani, R Domizi, AT Colesnicenco, E Montesi, S Ciucani, P Pelaia, C Ince

**Affiliations:** 1 Università Politécnica delle Marche, Ancona, Italy; 2 Academic Medical Center, Amsterdam, the Netherlands

## Introduction

Free hemoglobin (fHb) can scavenge nitric oxide and induce vasoconstriction [1]. The fHb content may be higher in older blood bags. We studied whether old red blood cell (RBC) transfusion increases plasma fHb in septic patients and if this affects the microvascular response.

## Methods

Twenty septic patients randomly received either fresh (<10 days storage) or old (>15 days) RBC transfusion. Plasma fHb was measured before and 1 hour after transfusion; the sublingual microcirculation was assessed with sidestream dark-field imaging. The perfused boundary region (PBR) was measured as an index of glycocalyx damage [2]. The thenar Tissue Hb index (THI) was measured (near-infrared spectroscopy).

## Results

**Figure 1:**
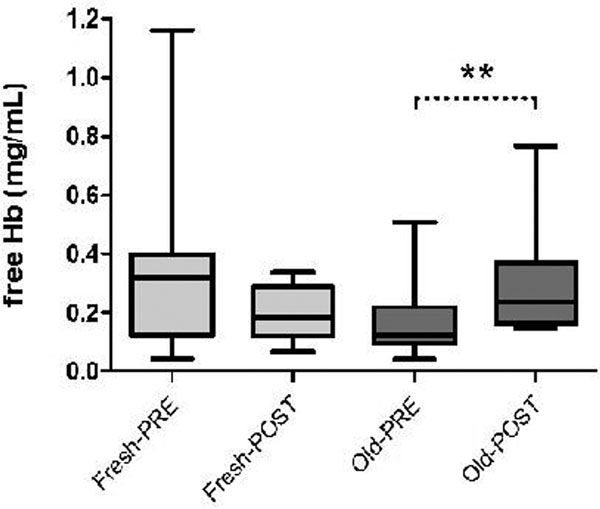
**Changes in fHb**.

**Figure 2:**
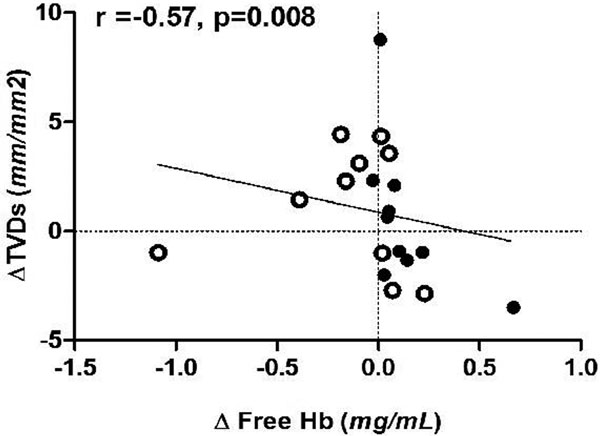
**Relation between changes in fHb and TVD**.

fHb increased in the old RBC group (Figure 1). THI increased in both groups, while SDF parameters were unaltered. Negative correlations were found between AfHb and changes in total vessel density (*r *= -0.57, *P *< 0.01; Figure 2) and THI (*r *= -0.71, *P *< 0.001). These relations were lacking in patients with PBR <2.68 μm.

## Conclusion

Old RBC transfusion increased plasma fHb in septic patients. Increasing plasma fHb levels after transfusion were associated with decreased microvascular density and lower increase in tissue Hb content. This relation might be blunted when the glycocalyx is preserved.
